# Autoimmune cytopenias (AIC) following allogeneic haematopoietic stem cell transplant for acquired aplastic anaemia: a joint study of the Autoimmune Diseases and Severe Aplastic Anaemia Working Parties (ADWP/SAAWP) of the European Society for Blood and Marrow Transplantation (EBMT)

**DOI:** 10.1038/s41409-019-0680-4

**Published:** 2019-09-25

**Authors:** Paul D. E. Miller, John A. Snowden, Regis Peffault De Latour, Simona Iacobelli, Diderik-Jan Eikema, Cora Knol, Judith C. W. Marsh, Carmel Rice, Mickey Koh, Franca Fagioli, Sridhar Chaganti, Jürgen Finke, Rafael F. Duarte, Peter Bader, Dominique Farge, Jakob R. Passweg, J. Alejandro Madrigal, Carlo Dufour

**Affiliations:** 1 Anthony Nolan Research Institute, London, UK; 2 Department of Haematology, Sheffield Teaching Hospitals NHS Foundation Trust, Sheffield, UK; 3 Department of Haematology, Hopital Saint-Louis, Paris, France; 4 Department of Biology, University of Rome Tor Vergata, Rome, Italy; 5 EBMT Statistical Unit, Leiden, Netherlands; 6 EBMT Data Office, Leiden, Netherlands; 7 Department of Haematological Medicine, Kings College Hospital, London, UK; 8 Department of Haematology, St George’s Hospital NHS Foundation Trust, London, UK; 9 Pediatric Onco-Hematology, Stem Cell Transplantation and Cellular Therapy Division, Regina Margherita Children’s Hospital, Torino, Italy; 10 Centre for Clinical Haematology, University Hospital Birmingham, Birmingham, UK; 11 Department of Hematology and Oncology, University Medical Center, Freiburg, Germany; 12 Department of Hematology, Hospital Universitario Puerta de Hierro, Madrid, Spain; 13 University Children’s Hospital Frankfurt, Frankfurt, Germany; 14 Department of Autoimmune Diseases and Vascular Pathology, Hopital Saint-Louis, Paris, France; 15 Division of Hematology, University Hospital of Santander, Basel, Switzerland; 16 Hematology Unit, G. Gaslini Children’s Hospital, Genova, Italy

**Keywords:** Epidemiology, Anaemia, Risk factors

## Abstract

This retrospective study explored the incidence of autoimmune cytopenia (AIC) in 530 paediatric and adult patients with acquired aplastic anaemia (aAA) who underwent first allogeneic HSCT between 2002 and 2012. AIC was a rare complication with a cumulative incidence of AIC at 1, 3, 5 and 10 years post HSCT of 2.5% (1.2–3.9 95% CI), 4.4% (2.6–6.2 95% CI), 4.6% (2.8–6.5 95% CI) and 5.1% (3.1–7.2 95% CI). Overall survival at 5 years after diagnosis of AIC was 85.9% (71–100 95% CI). Twenty-five patients were diagnosed with AIC at a median of 10.6 (2.6–91.5) months post HSCT. Eight (32%) patients were diagnosed with immune thrombocytopenia (ITP), seven (28%) with autoimmune haemolytic anaemia (AIHA), seven (24%) with Evans syndrome and four (16%) with autoimmune neutropenia (AIN). Treatment strategies were heterogeneous. Complete responses were seen in 12 of 25 patients, with death in three patients. In multivariable Cox analysis of a subgroup of 475 patients, peripheral blood stem cell (PBSC) transplant was associated with higher risk of AIC compared with bone marrow (BM) when conditioning regimens contained fludarabine and/or alemtuzumab (2.81 [1.06–7.49 95% CI]; *p* = 0.038), or anti-thymocyte globulin (ATG) (2.86 [1.11–7.37 95% CI]; *p* = 0.029). Myeloablative conditioning was associated with a lower risk of AIC compared with reduced intensity conditioning (RIC) in fludarabine and/or alemtuzumab (0.34 [0.12–0.98 95% CI]; *p* = 0.046) and ATG containing regimens (0.34 [0.12–0.95 95% CI]; *p* = 0.04). These findings provide clinically useful information regarding the incidence of a rare and potentially life-threatening complication of allogeneic HSCT for aAA, and further support for BM as the preferred stem cell source for transplant of patients with aAA.

## Introduction

Graft versus host disease (GvHD) describes alloreactivity between a transplanted donor immune system and host major or minor histocompatibility antigen, and is the principal immune mediated complication of allogeneic haematopoietic stem cell transplant (HSCT). The phenomenon of post-HSCT autoimmune disease (AID) is also recognized, and is the pathological autoreactivity towards non-histocompatibility antigen common to donor and recipient. Reported transmission of autoantibodies and AIDs from donor to recipient, and a case of immune thrombocytopenia (ITP) in the context of syngeneic HSCT suggest that this is a pathological entity distinct from GvHD [1, 2]. Whether autoreactivity develops exclusively in the graft, or also in host immune cells that have survived conditioning has not been determined. The most commonly encountered AIDs post HSCT are autoimmune cytopenias (AIC) [3]. AICs have previously been described in a number of HSCT contexts, but large registry studies have not explored AIC following HSCT for a single disease entity [4–9]. Acquired aplastic anaemia (aAA) is thought to be an autoimmune disorder in most cases [10] and we hypothesised that the incidence of post HSCT AIC in this population may be higher than described in other patient populations. We also sought to explore AIC diagnostic strategies, treatment approaches and patient outcomes across European Society for Blood and Marrow Transplantation (EBMT) centres.

## Methods

This study was conducted in accordance with the Declaration of Helsinki, and approved by the EBMT Autoimmune Disease Working Party and Severe Aplastic Anaemia Working Party. All EBMT centres performing allogeneic HSCT for aAA were invited to participate in the study. Centres agreeing to participate provided data on all consecutive paediatric and adult patients with aAA treated between January 2002 and December 2012 with first allogeneic HSCT. Centres confirmed whether AIC was diagnosed post-HSCT. Patients diagnosed with AIC at any time point prior to HSCT were excluded. Data for all patients were extracted from the EBMT registry, and centres identifying cases of AIC provided additional data on diagnostic criteria and investigations undertaken, therapies administered and patient outcome.

The primary study objective was to estimate the incidence of AIC diagnosed after allogeneic HSCT for aAA. Secondary objectives were to identify patient, donor and HSCT-related risk factors for development of AIC; to scope diagnostic criteria for AIC used across EBMT centres; to explore treatment strategies and response; and to estimate overall survival (OS) following onset of AIC.

ITP, autoimmune haemolytic anaemia (AIHA) and autoimmune neutropenia (AIN) were defined as a new or worsening thrombocytopenia with a platelet count of <100 × 10^9^/L, a new or worsening anaemia with a fall in haemoglobin of ≥20 g/L and new or worsening neutropenia <1.5 × 10^9^/L respectively. Centres were asked to indicate further laboratory features that had established an AIC diagnosis. Lines of treatment are presented as reported by participating centres. Complete response was defined as a normalisation of haemoglobin, platelet or neutrophil count and normalisation of biochemical markers of haemolysis for AIHA and Evans syndrome. Partial response was defined as an improvement but not normalisation, and no response as either no improvement or a decline in these parameters.

Pre-transplant patient characteristics were expressed as the median and range for continuous variables and frequencies and proportions for categorical variables. The primary endpoint was the incidence of post-transplant AIC, evaluated at 1, 3, 5 and 10 years after transplant. OS for the study cohort was evaluated at 1, 3, 5 and 10 years after transplant. OS after AIC diagnosis was analysed separately and evaluated at 5 years after AIC diagnosis. Median follow-up was determined using the reverse Kaplan–Meier method. OS was estimated using the Kaplan–Meier product limit estimation method, and differences in subgroups were assessed by the log-rank test. Cumulative incidences of AIC and death without AIC were analysed together in a competing risks framework. Subgroup differences were assessed using Gray’s test. The effect of specific conditioning regimens on the incidence of AIC and mortality without AIC were investigated using multivariable Cox models, providing cause specific hazards. The association between chronic GvHD and AIC was explored using a time dependent Cox model. A landmark analysis was used to compare incidence of AIC between those patients with and without acute GvHD. All reported estimates include 95% confidence intervals. All *p*-values were two-sided and *p* < 0.05 was considered significant. Statistical analyses were performed using SPSS version 22 (SPSS Inc., Chicago, IL) and R version 3.0.3.

## Results

A total of 530 patients (37.2% paediatric, 62.8% adult) from 41 participating centres were eligible for inclusion in the study. Median age at HSCT was 21.4 years (1.7–69.8). Median follow-up time was 6.4 years (interquartile range 4.2–9.0), and 1, 5 and 10-year OS for the cohort was 85.3% (82.3–88.4 95% CI), 80.8% (77.4–84.2 95% CI) and 78.8% (75.0–82.6 95% CI).

Characteristics of patients, grafts and conditioning regimens are provided in Table 1. Twenty-five patients were diagnosed with AIC at a median of 10.6 (2.6–91.5) months post allogeneic HSCT. Specific diagnoses were: 32.0% (*n* = 8) ITP, 28.0% (*n* = 7) AIHA, 24.0% (*n* = 6) Evans syndrome (five cases with AIHA and ITP, one case with AIHA and AIN) and 16.0% (*n* = 4) AIN (Table 2).

**Table 1:** Characteristics of *n* = 530 recipients of allogeneic HSCT for aAA between 2002 and 2012

	*n*	AIC cumulative incidence	95% CI	*p*-value
Patient sex				
Male	316	5.6	2.7–8.5	
Female	210	4.6	1.6–7.5	0.779
Patient age group				
<18	196	3.2	0.7–5.8	
≥18	331	6.4	3.3–9.4	0.209
Interval aAA diagnosis to treatment				
<12 months	379	4.5	2.4–6.7	
>12 months	148	6.6	1.8–11.3	0.589
Patient CMV status				
Negative	156	8.3	2.8–13.9	
Positive	311	3.7	1.6–5.9	0.142
Donor type				
Related	328	3.8	1.3–6.3	
Unrelated	191	7.5	3.7–11.3	0.077
Stem cell source				
Bone Marrow	358	3.3	1.2–5.6	
Peripheral Blood	141	9.7	4.7–14.8	
Cord Blood	20	5.0	0.0–14.6	0.01*
Patient/donor CMV match				
Matched	324	5.4	2.6–8.3	
Mismatched	134	4.7	1.0–8.3	0.959
Patient/donor sex match				
Matched	294	4.9	2.0–7.9	
Mismatched—male donor	109	5.8	1.3–10.3	
Mismatched—female donor	110	4.7	0.7–8.7	0.854
Conditioning type				
Myeloablative	297	2.9	0.6–5.2	
Reduced Intensity	198	8.1	4.2–12.1	0.005*
TBI				
Yes	53	8.1	4.8–15.7	
No	461	4.9	2.7–7.1	0.305
Fludarabine containing regimen				
Yes	307	6.5	3.7–9.4	
No	220	3.0	1.9–5.8	0.037*
Anti-thymocyte globulin containing regimen				
Yes	239	3.4	0.0–6.3	
No	288	6.7	3.7–9.6	0.04*
ATG and fludarabine	118	4.2	0.0–9.4	
ATG without fludarabine	121	2.7	0.0–5.6	0.969
Alemtuzumab containing regimen				
Yes	120	9.6	4.2–15.1	
No	407	3.7	1.6–5.7	0.007*
Alemtuzmab and fludarabine	99	10.7	4.4–17.0	
Alemtuzumab without fludarabine	21	4.8	0.0–13.9	0.408

^*^Statistically significant at 95% confidence level

**Table 2:** Treatment and outcome of 25 patients diagnosed with AIC following allogeneic HSCT for aAA

Case no.	Diagnosis	Gender	Age at allograft (Years)	Years from AlloHSCT to AIC diagnosis	DAT/autoantibody	Treatment line and response								Months from AIC diagnosis to last follow-up	AIC status at last follow-up
						First		Second		Third		Fourth			
1	ITP	Female	21	2.4	NA	No treatment								5.9	Untreated
2	ITP	Female	50	2.7	NA	No treatment								30.0	Untreated
3	ITP	Male	23	7.6	NA	Corticosteroid, IVIg	CR							10.2	CR (died)
4	ITP	Male	58	1.4	NA	CSA	PR*							46.0	PR
5	ITP	Male	21	0.6	NA	CSA, MMF, Rituximab	NR	Splenectomy	CR					64.7	CR
6	ITP	Female	37	1.2	NA	IVIg	CR	IVIg	CR					96.6	CR
7	ITP	Male	26	0.2	NA	Corticosteroid	NR	IVIg	NR	Splenectomy	CR			78.2	CR
8	ITP	Female	68	1.6	NA	Corticosteroid	NR	IVIg	NR	Rituximab	NR			5.9	NR (died)
9	AIHA	Male	38	0.5	IgG and C3d	Corticosteroid	CR							52.8	CR
10	AIHA	Female	17	0.4	IgG and C3d	Corticosteroid, IVIg	NR	Rituximab, PEX	CR					142.0	CR
11	AIHA	Male	17	1.4	IgG and C3d	Corticosteroid	NR	Rituximab	PR					6.28	PR
12	AIHA	Male	20	0.4	IgG	Corticosteroid, IVIg	NR	Rituximab, CSA	CR					MD	CR
13	AIHA	Male	42	0.7	Not performed	Corticosteroid	CR*	Corticosteroid	CR*					161.8	CR
14	AIHA	Male	7	0.6	IgG and C3d	Corticosteroid	CR	Rituximab	CR	Rituximab, MMF	CR	Rituximab	CR	69.4	CR
15	AIHA	Female	20	0.8	IgG and C3d	Corticosteroid	NR	Rituximab	NR	IVIG	NR	Splenectomy	CR	134.1	CR
16	Evans Syndrome (AIHA and ITP)	Female	5	2.4	IgG	Corticosteroid, IVIg	NR	Rituximab	NR					127.4	NR
17	Evans Syndrome (AIHA and ITP)	Female	18	0.4	IgG and C3d	Corticosteroid, IVIg	CR*	Corticosteroid, IVIg, Rituximab	CR*					36.3	CR
18	Evans Syndrome (AIHA and ITP)	Male	45	0.3	IgG	Corticosteroid, Rituximab	CR	Corticosteroid, IVIg	PR					81.4	PR
19	Evans Syndrome (AIHA and ITP)	Male	19	0.4	IgG	Corticosteriod, IVIg	PR*	Corticosteroid, IVIg	NR	IVIg	PR*			164.9	PR
20	Evans syndrome (AIHA and AIN)	Male	22	0.5	IgG and C3d	Corticosteroid, Rituximab	NR	Cyclophosphamide, PEX	NR	Splenectomy	NR			1.0	NR (died)
21	Evans Syndrome (AIHA and ITP)	Female	11	1.0	IgG	IVIg	NR	Rituximab	NR	MMF	NR	Splenectomy	PR	52.7	PR
22	AIN	Female	4	Unknown	ANAb detected	No treatment								MD	Untreated
23	AIN	Male	25	4.4	ANAb not detected	No treatment								MD	Untreated
24	AIN	Male	63	1.2	ANAb not detected	GCSF	PR*							36.6	PR
25	AIN	Male	55	1.0	ANAb not detected	GCSF	PR*	GCSF, Rituximab	CR*					17.6	CR

*NR* no response, *PR* partial response, *CR* complete response, *ANAb* anti-neutrophil antibody, *CSA* ciclosporin A, *GCSF* granulocyte-colony-stimulating factor, *IVIG* intravenous immunoglobulin, *MMF* mycophenolate mofetil, *PEX* plasma exchange, *NA* not applicable, *MD* missing data

*Maintenance therapy required

The cumulative incidence of AIC at 1, 3, 5 and 10 years post HSCT was 2.5% (1.2–3.9 95% CI), 4.4% (2.6–6.2 95% CI), 4.6% (2.8–6.5 95% CI) and 5.1% (3.1–7.2 95% CI), respectively. OS at 5 years after diagnosis of AIC was 85.9% (71–100 95% CI), with all deaths occurring within the first 12 months.

### Diagnostic criteria

As the study was retrospective, central review of peripheral blood smears and bone marrow examination (BME) samples was not possible. In addition to reporting diagnostic criteria, centres were asked to report whether all differential diagnoses had been excluded. It was decided a priori to include all reported cases of AIC in study analysis, and describe the diagnostic features employed by participating centres in order to present real-world practice during the study period. For ITP, all patients had a new or worsening thrombocytopenia with a platelet count of <100 × 10^9^/L. BME was performed in 6/8 patients, with four displaying normo- or hyper-regenerative megakaryopoiesis, and two other morphological or histological findings. Five centres indicated that differential diagnoses (disease relapse, new haematological malignancy, drug induced thrombocytopenia, infection, thrombotic microangiopathy and alloimmune thrombocytopenia) had been excluded, two centres reported that this was unknown and one that all differential diagnoses had not been excluded. All AIHA patients had a new or worsening anaemia with a fall in haemoglobin of ≥20 g/L. Direct antiglobulin test (DAT) was performed in 6/7 cases with one IgG positive, and five IgG and C3d positive (Table 2). All centres identified at least one biochemical marker of haemolysis (raised lactate dehydrogenase, unconjugated hyper-bilirubinaemia or reduced haptoglobin) and three identified a peripheral blood reticulocytosis. Three centres indicated that differential diagnoses had been excluded (disease relapse, new haematological malignancy, drug induced haemolysis, infection, microangiopathic haemolytic anaemia and alloimmune haemolytic anaemia), three reported this information was unknown and one that all differential diagnoses had not been excluded. Paired donor–recipient ABO blood group compatibility data were not complete for any of the patients diagnosed with AIHA. All cases of Evans syndrome were DAT positive (4 IgG, 2 IgG and C3d) and all cases involving AIHA and ITP had BME with four displaying normo- or hyper-regenerative megakaryopoiesis. Biochemical markers of haemolysis were present in 5/6 cases and two patients had a peripheral blood reticulocytosis. Three centres indicated that differential diagnoses (as for AIHA and ITP or AIN accordingly) had been excluded, two that this information was unknown and one that all differential diagnoses had not been excluded. Anti-neutrophil antibodies (ANAb) were screened for by either direct or indirect method in all AIN cases, and also in the Evans syndrome case presenting with AIHA and AIN. Antibodies were only detected in one patient. Three centres reported that differential diagnoses (disease relapse, new haematological malignancy, infection and drug induced neutropenia) had been excluded and one reported these data are unknown.

### Treatment and response

Of 25 patients diagnosed with AIC, 21 (84%) were treated, while 2 (8%) ITP (Cases 1 and 2) and 2 (8%) AIN patients (Cases 22 and 23) received no AIC specific therapy (Table 2). Treatment approach was heterogeneous regarding both choice of therapeutic agents, and number of agents used at each treatment line (Table 2). The mainstays of therapy for ITP, AIHA and Evans syndrome were corticosteroids and intravenous immunoglobulin (IVIg). Corticosteroids were administered to 50%, 100 and 83% of treated ITP, AIHA and Evans syndrome patients, respectively. IVIg was administered concurrently with corticosteroids, or as first or second-line monotherapy, in 66.7, 42.9 and 83.3% of ITP, AIHA and Evans syndrome patients. Rituximab was administered to 71.4% and 83.3% of patients with AIHA and Evans syndrome, respectively, but only 16.7% of ITP patients. Other immunomodulatory therapies for this group of patients were mycophenolate mofetil 15%, and cyclosporine A 15%. One patient with Evans syndrome presenting with AIHA and AIN (Case 20) received cyclophosphamide and underwent plasma exchange. Four (19.0%) patients did not respond or relapsed following immunomodulatory therapy and underwent splenectomy, with two achieving CR (ITP Case 7, AIHA Case 15), one PR (Evans syndrome Case 21) and one patient dying without responding (AIHA and AIN Case 20). Of the four patients with AIN, two (Cases 24 and 25) were treated with granulocyte-colony-stimulating factor to PR, one of whom required second-line therapy and achieved CR with rituximab (Case 25).

After excluding patients who did not require treatment, 10 out of this subgroup of 21 patients did not respond to first-line therapy, with 7 of 17, and 4 of 7 not responding to second or third-line therapy, respectively. Of the 11 of 21 treated patients who achieved either complete or partial response to first-line therapy, nearly two-thirds (7/11) relapsed and required further treatment. AIC status at last treatment was 4/25 untreated, 12/25 CR, 6/25 in PR and 3/25 NR. Six out of twenty one treated patients required on-going maintenance therapy (Table 2). Highest CR rate at last follow-up was seen in AIHA (6/7 patients) and lowest in Evans syndrome (1/7). Incidence of mortality was 13% (0–26% 95% CI) at 2 years, with one death in CR from a non-AIC HSCT-related complication (Case 3, Table 2) and two without responding to AIC therapy who both died from infection (Cases 8 and 20, Table 2).

### Risk factors for development of AIC

In univariable analysis, cumulative incidence of AIC at 10 years post HSCT did not vary with patient sex, patient age group, interval from aAA diagnosis to HSCT, patient cytomegalovirus (CMV) status, donor type, patient-donor CMV match, patient-donor sex match or conditioning with TBI (Table 1). Of 191 unrelated donor–recipient pairs, 61 were reported as fully human leukocyte antigen (HLA) matched, however HLA data were unavailable for the remaining 110 and so further analysis could not be performed. The overall incidence of grade II–IV aGvHD was 13% (9–16 95% CI). There was no difference in 10-year incidence of AIC between patients with and without aGVHD (2% [0–5 95% CI] vs 5% [2–7 95% CI], *p* = 0.3). The incidence of limited and extensive cGvHD was 20% (16–24 95% CI) at both 60 and 120 months. There was no association between cGvHD and development of AIC (Hazard ratio 0.6 [0.14–7 95% CI], *p* = 0.66). We did not evaluate the temporal relationship between withdrawal of immunosuppression and onset of AIC. Ten-year cumulative incidence of AIC was higher amongst patients treated with non-myeloablative conditioning (MAC) compared with MAC (8.1% [4.2–12.1 95% CI] vs 2.9% [0.6–5.2 95% CI], *p* = 0.005), with conditioning regimens containing fludarabine compared with regimens not containing fludarabine (6.5% [3.7–9.4 95% CI] vs 3.0% [1.9–5.8 95% CI], *p* = 0.037) and with alemtuzumab containing regimens compared with non-Alemtuzumab containing regimens (9.6% [4.2–15.1 95% CI] vs 3.7% [1.6–5.7 95% CI], *p* = 0.007). Conditioning with anti-thymocyte globulin (ATG) was associated with a lower incidence of AIC than conditioning without ATG (3.4% [0.0–6.3 95% CI] vs 6.7% [3.7–9.6 95% CI], *p* = 0.04). Incidence of AIC was also significantly higher in patients receiving peripheral blood stem cell (PBSC) (9.7% [4.7–14.8 95% CI]) compared with umbilical cord blood (UCB) (5.0% [0.0–14.6 95% CI]) and bone marrow (BM) (3.3% [1.2–5.6 95% CI]) (*p* = 0.01). Incidence of AIC was not higher in regimens containing alemtuzumab and fludarabine compared with alemtuzumab alone (10.7% [4.4–17.0 95% CI] vs 4.8% [0–13.9 95% CI]; *p* = 0.408), nor in regimens containing ATG and fludarabine compared with ATG alone (4.2% [0.0–9.6 95% CI] vs 2.7% [0.0–5.6 95% CI]; *p* = 0.969).

A subgroup of 475 patients who received either BM or PBSC grafts and had complete information on conditioning regimen were included in a multivariable analysis. Two models explored the independent association of conditioning intensity, stem cell source, use of alemtuzumab and/or fludarabine or use of ATG with risk of AIC (Table 3). In the alemtuzumab and/or fludarabine model, these agents were not associated with an increased risk of AIC (1.25 [0.37–4.19 95% CI]; *p* = 0.723). However, after adjustment for alemtuzumab and/or fludarabine, transplant with PBSC remained independently associated with a higher risk of AIC (2.81 [1.06–7.49 95% CI]; *p* = 0.038), and MAC with lower risk (0.34 [0.12–0.98 95% CI]; *p* = 0.046). Likewise, in the multivariable ATG model, use of this serotherapy in conditioning was not associated with higher risk of AIC (0.55 [0.21–1.41 95% CI]; *p* = 0.212) but PBSC (2.86 [1.11–7.37 95% CI]; *p* = 0.029) and MAC (0.34 [0.12–0.95 95% CI]; *p* = 0.04) remained independently associated with higher and lower risk, respectively. There was no increase in the competing risk, death without AIC, associated with any of these variables. Owing to small number of AIC cases it was not possible to adjust for additional characteristics and so these findings are not generalizable beyond the study population.

**Table 3:** Estimated cause specific hazard ratios (sHR) for AIC and death without AIC using multivariable regression model for *n* = 475 recipients of allogeneic HSCT for aAA between 2002 and 2012

Risk factor	Group	*n*	AIC			Death without AIC		
			events	sHR (95% CI)	*p*	events	sHR (95% CI)	*p*
	Total	475	21			89		
Alemtuzumab and fludarabine	No alemtuzumab and no fludarabine	178	4			25		
	Alemtuzumab and/or fludarabine	297	17	1.25 (0.37–4.19)	0.723	64	1.4 (0.85–2.31)	0.181
Stem cell source	BM	340	8			58		
	PB	135	13	2.81 (1.06–7.49)	0.038*	31	1.15 (0.71–1.85)	0.572
Myeloablative conditioning	No	186	15			42		
	Yes	289	6	0.34 (0.12–0.98)	0.046*	47	0.77 (0.49–1.22)	0.272
ATG	No ATG	267	15			45		
	ATG	208	6	0.55 (0.21–1.41)	0.212	44	1.18 (0.78–1.79)	0.438
Stem cell source	BM	340	8			58		
	PB	135	13	2.86 (1.11–7.37)	0.029*	31	1.26 (0.79–2)	0.338
Myeloablative conditioning	No	186	15			42		
	Yes	289	6	0.34 (0.12–0.95)	0.04*	47	0.72 (0.46–1.12)	0.14

*Statistically significant at 95% confidence level

## Discussion

Ten-year cumulative incidence of AIC after allogeneic HSCT for aAA was 5.1% (3.1–7.2 95% CI). ITP was the most frequent AIC diagnosis, followed by AIHA, Evans and AIN. BME was not performed in two patients diagnosed with ITP. In a further two patients BME did not demonstrate normo- or hyper-regenerative megakaryopoiesis. It is possible that these patients were misdiagnosed biasing our results. AIC complicated both the early and late post-HSCT period, with time of diagnosis ranging from 2.6 months to 7.6 years. Incidence of AIC in our cohort is similar to that reported in previous studies of HSCT populations. In paediatric patients transplanted with a range of allogeneic graft types for malignant and non-malignant conditions, a 2.4–2.5% 10-year incidence of AIC, and a 2.4% incidence of AIHA are reported [6, 8]. Among adult recipients of UCB HSCT for haematological malignancy, 3 year incidence of AIHA and ITP were 5.4% and 1.4%, respectively [4]. Incidence of AIHA was similar at 4.1–4.4% amongst adult recipients transplanted with a range of graft types [7, 11]. Median time of diagnosis in these studies ranged from 0.8 months to 8.4 years.

Cases of AIC in our study were diagnosed in accordance with recognized criteria [12–14]. In the absence of haematological or biochemical markers of platelet destruction, ITP is a diagnosis of exclusion requiring thorough assessment. However BME is recommended only in selected paediatric and adult patients, generally those in whom secondary ITP is suspected, or adults over 60 [15, 16]. In our present study, most patients diagnosed with ITP and all with Evans syndrome underwent BME, which may reflect the need to exclude HSCT specific differentials including disease relapse and graft failure. While all cases of AIHA, and all but one case of Evans syndrome presented with at least one abnormal biochemical marker of haemolysis, reticulocytosis was not a dominant feature present in one-third of AIHA and Evans syndrome patients. Testing for ANAb is recommended as part of neutropenia work-up, however antibodies are typically of low titre and low avidity and testing of rather limited sensitivity and specificity [14, 17]; thus a negative ANAb screen does not exclude a diagnosis of AIN. In line with this, all cases of AIN were tested for ANAb but the majority were negative.

Most ITP patients were treated with corticosteroid or IVIg, while rituximab was infrequently used. Fifty percent of ITP patients achieved either CR or PR with each line of therapy. In the general population a response to first-line steroids of up to 80% has been reported [16, 18], and the combination of IVIg with corticosteroid therapy may hasten platelet recovery [19]. In randomized studies, the combination of rituximab with dexamethasone compared with dexamethasone alone has been shown to improve response rates from 36–37 to 58–63% [20, 21]. Case reports and series of patients with ITP post HSCT have reported response rates of 30–50% following first and second-line treatment, with the former largely consisting of corticosteroid with or without IVIg, and the latter IVIg, rituximab or splenectomy [22–26]. In cases of post-HSCT ITP refractory to standard therapy, thrombopoietin receptor agonists (TPO-RAs) have proven effective [27–29]. In our cohort no patients were treated with TPO-RAs, which may reflect European Medicines Agency licensing of romiplostim in 2009 and eltrombopag in 2010, during the last 3 years of our study eligibility period.

Although randomized data are lacking, corticosteroids are an established first-line therapy for AIHA with retrospective studies reporting responses of ~75% [30]. Again, there is limited evidence for second-line therapy, but rituximab or splenectomy are the most commonly described [31]. Similar strategies are reported in the management of paediatric and adult Evans syndrome [32, 33]. Patients in our study were largely treated according to this paradigm: all patients with AIHA received first-line corticosteroid with or without IVIg. One patient requiring second-line therapy was re-treated with corticosteroids; and all others received second-line rituximab with all patients achieving a CR or PR. Evans syndrome appeared the most challenging AIC to treat, with the greatest proportion of non-responders. However, there was no excess of deaths in this group compared with other AIC diagnoses. In cases series of patients with AIHA post-HSCT, 14–33% response rates are reported with prednisolone alone and 40–45% with prednisolone in combination with rituximab or IVIg [6–9, 26, 34]. In these series, up to 33% of patients were reported refractory to therapy, whereas all patients in our series were in CR or PR at last follow-up.

Primary AIN is typically a disease of infancy, while secondary AIN occurs mostly in adults in the context of systemic AID [35]. Neutropenia may respond to treatment of the underlying AID with GCSF administered cautiously owing to concern about disease flare [14, 17]. Case reports of AIN post HSCT have described disease ranging from transient positive ANAb and neutropenia not requiring treatment [36] to persistent neutropenia requiring GCSF support alone [5] or in combination with additional immunomodulatory agents [6]. This pattern of disease is borne out in our study with half of diagnosed patients requiring no specific therapy. Two were treated with GCSF, one of whom required additional therapy with rituximab. Both treated patients achieved a PR.

The pathogenesis of AID remains poorly understood but relates to breakdown in mechanisms of self-tolerance. First, there may be a genetic role, with documented familial aggregation of autoimmune conditions [37], the association of certain HLA types with AIDs [38] and autoimmunity manifesting in inherited primary immunodeficiency [39]. Secondly, environmental factors may contribute; in murine models inflammatory states have been show to trigger and promote autoreactive T-cell populations [40, 41], and infection may be associated with lymphodepletion and homeostatic expansion favouring autoreactive clones [42]. Finally, iatrogenic dysregulation of central and peripheral immune tolerance through radio-, chemo- or immunotherapy may lead to impaired thymic function, lymphodepletion and homeostatic expansion and failure of regulatory T-cell control. Although the pathogenesis of secondary AIDs post HSCT also remains unclear, these factors may play a role. In univariable analysis we identified an increased incidence of AIC amongst patients treated with alemtuzumab or fludarabine. These two drugs have been associated with autoimmune phenomenon in a range of therapeutic settings [43–51] and in a cohort of aAA HSCT patients conditioned with fludarabine and alemtuzumab 14% of patients developed autoimmune complications [52]. In contrast with our findings, conditioning with ATG has been associated with an increased risk of secondary autoimmune phenomenon following autologous HSCT for primary AID [45, 46], with lymphocyte depletion the putative mechanism. These findings did not remain significant in multivariable analysis, possibly due to small number of events in subgroup analysis, and further investigation is warranted. An association between post-HSCT AID and cGvHD has been reported, although inconsistently [4, 6–8, 26]. In this study an association between AIC and GvHD was not identified.

In allogeneic HSCT for aAA, BM conveys a lower risk of GvHD and a survival advantage compared with PBSC, and is the preferred stem cell source in all age groups and in recipients of sibling and unrelated donor transplants [53–56]. Nonetheless, approximately one-third of all patients were transplanted with either PBSC or UCB in our study. Incidence of AIC was significantly higher amongst recipients of PBSC compared with UCB and BM. In multivariable analysis, after adjustment for concurrent ATG, and fludarabine and/or alemtuzumab, transplant with PBSC and reduced intensity conditioning (RIC) was independently associated with a higher risk of AIC. A higher number of immunocompetent cells are harvested and transplanted with PBSC compared with BM [57], and immune recovery is more rapid [58]. Early T-cell recovery occurs predominantly through peripheral expansion, rather than de novo thymic ontogenesis in both T replete and deplete grafts [59, 60]. In RIC, host T cells that survive the conditioning regimen may also expand through this mechanism [61]. Such homeostatic expansion contributes to a narrow T-cell repertoire, qualitative immune deficiencies [61] and may favour autoreactive clones [42], contributing to the emergence of AID [62]. It is possible that the larger number of immunocompetent cells and more rapid immune reconstitution in PBSC compared with BM, and the potential for homeostatic expansion of host lymphocytes in RIC promotes development of autoimmunity; immune reconstitution studies in patients with AIC may provide further detail.

This is the first study to explore AIC following allogeneic HSCT in a large cohort for a single disease indication, and provides clinically useful information on the incidence of this rare and potentially life-threatening complication in paediatric and adult patients. The retrospective study design may impact on our estimate of AIC incidence: cases may not have been reported and we were unable to confirm AIC diagnoses through central review of pathology. For eight patients with AIC, centres reported that it was unknown whether all differential diagnoses had been excluded, and for 3 that all differentials had not been excluded. These cases were included in analysis to reflect real-world practice and highlight the challenging nature of establishing these diagnoses, but it remains possible that these patients were misdiagnosed biasing our results. As there were relatively few cases of AIC, we were able to develop a multivariable model with a small number of variables that limits generalisability of our findings. We did not explore the role of pre-transplantation therapy on risk of developing AIC.

## Conclusions

In conclusion, the incidence of AIC post allogeneic HSCT for aAA is similar to that reported among recipients of HSCT for other primary diseases. Autoimmunity should be considered in the differential diagnosis of cytopenias occurring in both the early and late post HSCT period. Our study highlights heterogeneity in the approach to investigation and management of AIC between EBMT centres. As such standardization of diagnostic and therapeutic approaches, along with data registration (via the EBMT and other databases), is warranted to inform harmonized recommendations for investigation and management of suspected AIC. Response rates in this cohort are similar to previous data and OS in the AIC group was not shorter than in the overall cohort. In multivariable analysis we identified an increased risk of AIC following PBSC and RIC HSCT in this cohort, which may contribute further to the evidence that BM is the preferred stem cell source for transplant of patients with aAA.

## References

[1] Nishio M, Sawada K, Koizumi K, Endoh T, Takashima H, Hashimoto H, et al. Autoimmune thrombocytopenia following syngeneic peripheral blood stem cell transplantation. Rinsho Ketsueki. 1998;39:580–5. http://www.ncbi.nlm.nih.gov/pubmed/9785976. 9785976

[2] Niederwieser D, Gentilini C, Hegenbart U, Lange T, Moosmann P, Pönisch W.et al. Transmission of donor illness by stem cell transplantation: should screening be different in older donors?. Bone Marrow Transpl. 2004;34:657–65. http://www.nature.com/articles/1704588. 10.1038/sj.bmt.1704588 15334048

[3] Holbro A, Abinun M, Daikeler T. Management of autoimmune diseases after haematopoietic stem cell transplantation. Br J Haematol. 2012;157:281–90. http://ovidsp.ovid.com/ovidweb.cgi?T=JS&PAGE=reference&D=emed10&NEWS=N&AN=2012204407. 10.1111/j.1365-2141.2012.09070.x 22360687

[4] Sanz J, Arango M, Carpio N, Montesinos P, Moscardó F, Martín G, et al. Autoimmune cytopenias after umbilical cord blood transplantation in adults with hematological malignancies: a single-center experience. Bone Marrow Transpl. 2014;49:1084–8. http://www.ncbi.nlm.nih.gov/pubmed/24887383. 10.1038/bmt.2014.107 24887383

[5] Page KM, Mendizabal AM, Prasad VK, Martin PL, Parikh S.Wood S,et al. Posttransplant autoimmune hemolytic anemia and other autoimmune cytopenias are increased in very young infants undergoing unrelated donor umbilical cord blood transplantation. Biol Blood Marrow Transpl. 2008;14:1108–17. http://www.pubmedcentral.nih.gov/articlerender.fcgi?artid=3735356&tool=pmcentrez&rendertype=abstract. 10.1016/j.bbmt.2008.07.006 PMC3735356 18804040

[6] Faraci M, Zecca M, Pillon M, Rovelli A, Menconi MC, Ripaldi M.et al. Autoimmune hematological diseases after allogeneic hematopoietic stem cell transplantation in children: an Italian multicenter experience. Biol Blood Marrow Transplant. 2014;20:272–8. http://www.ncbi.nlm.nih.gov/pubmed/24274983. 10.1016/j.bbmt.2013.11.014 24274983

[7] Sanz J, Arriaga F, Montesinos P, Orti G, Lorenzo I, Cantero S.et al. Autoimmune hemolytic anemia following allogeneic hematopoietic stem cell transplantation in adult patients. Bone Marrow Transplant. 2007;39:555–61. http://www.ncbi.nlm.nih.gov/pubmed/17351645. 10.1038/sj.bmt.1705641 17351645

[8] Ahmed I, Teruya J, Murray-Krezan C, Krance R (2015). The incidence of autoimmune hemolytic anemia in pediatric hematopoietic stem cell recipients post first and second hematopoietic stem cell transplant HHS Public Access. Pediatr Transplant.

[9] Wang M, Wang W, Abeywardane A, Adikarama M, McLornan D, Raj K, et al. Autoimmune hemolytic anemia after allogeneic hematopoietic stem cell transplantation: analysis of 533 adult patients who underwent transplantation at King’s College Hospital. Biol Blood Marrow Transplant. 2015;21:60–6. http://linkinghub.elsevier.com/retrieve/pii/S1083879114005643. 10.1016/j.bbmt.2014.09.009 25262883

[10] Goto M, Kuribayashi K, Takahashi Y, Kondoh T, Tanaka M, Kobayashi D.et al. Identification of autoantibodies expressed in acquired aplastic anaemia. Br J Haematol. 2013;160:359–62. http://www.ncbi.nlm.nih.gov/pubmed/23116149. 10.1111/bjh.12116 23116149

[11] Zhen Y, Bangzhao W, Youning Z, Wenjuan W, Suning C, Aining S, et al. Clinical and serological characterization of autoimmune hemolytic anemia after allogeneic hematopoietic stem cell transplantation. Chin Med J. 2014;127:1235–8. http://www.cmj.org/ch/reader/create_pdf.aspx?file_no=20132823&year_id=2014&quarter_id=7&falg=1%5Cnhttp://ovidsp.ovid.com/ovidweb.cgi?T=JS&PAGE=reference&D=emed11&NEWS=N&AN=2014240199. 24709172

[12] Rodeghiero F, Stasi R, Gernsheimer T, Michel M, Provan D, Arnold DM.et al. Standardization of terminology, definitions and outcome criteria in immune thrombocytopenic purpura of adults and children: report from an international working group. Blood. 2009;113:2386–93. http://www.ncbi.nlm.nih.gov/pubmed/19005182. 10.1182/blood-2008-07-162503 19005182

[13] Hill QA, Stamps R, Massey E, Grainger JD, Provan D, Hill A. The diagnosis and management of primary autoimmune haemolytic anaemia. Br J Haematol. 2017;176:395–411. 10.1111/bjh.14478. 10.1111/bjh.14478 28005293

[14] Fioredda F, Calvillo M, Bonanomi S, Coliva T, Tucci F, Farruggia P, et al. Congenital and acquired neutropenia consensus guidelines on diagnosis from the Neutropenia Committee of the Marrow Failure Syndrome Group of the AIEOP (Associazione Italiana Emato-Oncologia Pediatrica). Pediatr Blood Cancer 2011;57:10–7. 10.1002/pbc.23108. 10.1002/pbc.23108 21448998

[15] Neunert C, Lim W, Crowther M, Cohen A, Solberg L, Crowther MA. The American Society of Hematology 2011 evidence-based practice guideline for immune thrombocytopenia. Blood. 2011;117:4190–207. http://www.bloodjournal.org/content/bloodjournal/117/16/4190.full.pdf. 10.1182/blood-2010-08-302984 21325604

[16] Provan D, Stasi R, Newland AC, Blanchette VS, Bolton-Maggs P, Bussel JB.et al. International consensus report on the investigation and management of primary immune thrombocytopenia. Blood. 2010;115:168–86. http://www.ncbi.nlm.nih.gov/pubmed/19846889. 10.1182/blood-2009-06-225565 19846889

[17] Youinou P, Jamin C, Le Pottier L, Renaudineau Y, Hillion S, Pers J-O. Diagnostic criteria for autoimmune neutropenia. Autoimmun Rev. 2014;13:574–6. http://www.sciencedirect.com/science/article/pii/S1568997214000111. 10.1016/j.autrev.2014.01.001 24418296

[18] Cheng G. Eltrombopag, a thrombopoietin- receptor agonist in the treatment of adult chronic immune thrombocytopenia: a review of the efficacy and safety profile. Ther Adv Hematol. 2012;3:155–64. http://www.ncbi.nlm.nih.gov/pubmed/23556122. 10.1177/2040620712442525 PMC3573439 23556122

[19] Godeau B, Chevret S, Varet B, Lefrère F, Zini JM, Bassompierre F.et al. Intravenous immunoglobulin or high-dose methylprednisolone, with or without oral prednisone, for adults with untreated severe autoimmune thrombocytopenic purpura: a randomised, multicentre trial. Lancet. 2002;359:23–9. http://linkinghub.elsevier.com/retrieve/pii/S0140673602072756. 10.1016/S0140-6736(02)07275-6 11809183

[20] Gudbrandsdottir S, Birgens HS, Frederiksen H, Jensen BA, Jensen MK, Kjeldsen L.et al. Rituximab and dexamethasone vs dexamethasone monotherapy in newly diagnosed patients with primary immune thrombocytopenia. Blood. 2013;121:1976–81. http://www.ncbi.nlm.nih.gov/pubmed/23293082. 10.1182/blood-2012-09-455691 23293082

[21] Zaja F, Baccarani M, Mazza P, Bocchia M, Gugliotta L, Zaccaria A, et al. Dexamethasone plus rituximab yields higher sustained response rates than dexamethasone monotherapy in adults with primary immune thrombocytopenia. Blood. 2010;115:2755–62. http://www.ncbi.nlm.nih.gov/pubmed/20130241. 10.1182/blood-2009-07-229815 20130241

[22] Hequet O, Salles G, Ketterer N, Espinouse D, Dumontet C, Thieblemont C.et al. Autoimmune thrombocytopenic purpura after autologous stem cell transplantation. Bone Marrow Transpl. 2003;32:89–95. http://www.ncbi.nlm.nih.gov/pubmed/12815483. 10.1038/sj.bmt.1704073 12815483

[23] Ahmad I, Haider K.Kanthan R, Autoimmune thrombocytopenia following tandem autologous peripheral blood stem cell transplantation for refractory germ cell tumor. Bone Marrow Transpl. 2004;34:279–80. http://www.ncbi.nlm.nih.gov/pubmed/15170159. 10.1038/sj.bmt.1704574 15170159

[24] Raj K, Narayanan S, Augustson B, Ho A, Mehta P, Duncan N.et al. Rituximab is effective in the management of refractory autoimmune cytopenias occurring after allogeneic stem cell transplantation. Bone Marrow Transpl. 2005;35:299–301. http://www.ncbi.nlm.nih.gov/pubmed/15568036. 10.1038/sj.bmt.1704705 15568036

[25] Fujita H, Togami K, Mori M, Hashimoto H, Nagai K, Nagai Y.et al. Successful treatment with azathioprine for autoimmune thrombocytopenia developing after autologous peripheral blood stem cell transplantation. Rinsho Ketsueki. 2007;48:637–41. http://www.ncbi.nlm.nih.gov/pubmed/17867300. 17867300

[26] Daikeler T, Labopin M, Ruggeri A, Crotta A, Abinun M, Hussein AA.et al. New autoimmune diseases after cord blood transplantation: a retrospective study of EUROCORD and the Autoimmune Disease Working Party of the European Group for Blood and Marrow Transplantation. Blood. 2013;121:1059–64. http://www.ncbi.nlm.nih.gov/pubmed/23247725. 10.1182/blood-2012-07-445965 23247725

[27] Beck JC, Burke MJ, Tolar J. Response of refractory immune thrombocytopenia after bone marrow transplantation to romiplostim. Pediatr Blood Cancer. 2010;54:490–1. http://www.ncbi.nlm.nih.gov/pubmed/19908296. 10.1002/pbc.22332 19908296

[28] Poon LM, Di Stasi A, Popat U, Champlin RE, Ciurea SO. Romiplostim for delayed platelet recovery and secondary thrombocytopenia following allogeneic stem cell transplantation. Am J Blood Res. 2013;3:260–4. http://www.pubmedcentral.nih.gov/articlerender.fcgi?artid=3755526&tool=pmcentrez&rendertype=abstract. PMC3755526 23997988

[29] O’Donovan EM, Rezvani K, Sargent J, Richardson D, Tharmalingam H, Veys P.et al. Thrombopoietic agonists show efficacy in ITP related to allogeneic stem cell transplantation. Blood. 2011;118:3292. http://www.bloodjournal.org/content/118/21/3292?sso-checked=true.

[30] Barcellini W, Fattizzo B, Zaninoni A, Radice T, Nichele I, Di Bona E, et al. Clinical heterogeneity and predictors of outcome in primary autoimmune hemolytic anemia: a GIMEMA study of 308 patients. Blood. Am Soc Hematol. 2014;124:2930–6. http://www.ncbi.nlm.nih.gov/pubmed/25232059. 10.1182/blood-2014-06-583021 25232059

[31] Crowther M, Chan YLT, Garbett IK, Lim W, Vickers MA, Crowther MA. Evidence-based focused review of the treatment of idiopathic warm immune hemolytic anemia in adults. Blood. Am Soc Hematol. 2011;118:4036–40. http://www.ncbi.nlm.nih.gov/pubmed/21778343. 10.1182/blood-2011-05-347708 21778343

[32] Michel M, Chanet VR, Dechartres AS, Morin A-S, Piette J-C, Cirasino L.et al. The spectrum of Evans syndrome in adults: new insight into the disease based on the analysis of 68 cases. Blood. 2009;114:3167–72. http://www.bloodjournal.org/content/bloodjournal/114/15/3167.full.pdf?sso-checked=true. 10.1182/blood-2009-04-215368 19638626

[33] Miano M. How I manage Evans syndrome and AIHA cases in children. Br J Haematol. 2016;172:524–34. http://www.ncbi.nlm.nih.gov/pubmed/2662587. 10.1111/bjh.13866 26625877

[34] Chen F, Owen I, Savage D (1997). Late onset haemolysis and red cell autoimmunisation after allogeneic bone marrow transplant. Bone Marrow Transpl.

[35] Gibson C, Berliner N (2014). How we evaluate and treat neutropenia in adults. Blood.

[36] Tosi P, Bandini G, Tazzari P, Raspadori D, Cirio TM, Rosti G.et al. Autoimmune neutropenia after unrelated bone marrow transplantation. Bone Marrow Transpl. 1994;14:1003–4. http://www.ncbi.nlm.nih.gov/pubmed/7711662. 7711662

[37] Cárdenas-Roldán J, Rojas-Villarraga A, Anaya J-M. How do autoimmune diseases cluster in families? A systematic review and meta-analysis. BMC Med. 2013;11:1–22. http://www.ncbi.nlm.nih.gov/pubmed/23497011. 10.1186/1741-7015-11-73 PMC3655934 23497011

[38] Gough SCL, Simmonds MJ. The HLA region and autoimmune disease: associations and mechanisms of action. Curr Genom. 2007;8:453–65. http://www.ncbi.nlm.nih.gov/pubmed/19412418. 10.2174/138920207783591690 PMC2647156 19412418

[39] Consolini R, Renee Forbes L, Wahlstrom J, Pignata C, Giardino G, Gallo V (2016). Unbalanced immune system: immunodeficiencies and autoimmunity. Front Paediatr.

[40] Hirota K, Hashimoto M, Yoshitomi H, Tanaka S, Nomura T, Yamaguchi T.et al. T cell self-reactivity forms a cytokine milieu for spontaneous development of IL-17 + Th cells that cause autoimmune arthritis. J Exp Med. 2007;204:41–7. http://www.ncbi.nlm.nih.gov/pubmed/1722791. 10.1084/jem.20062259 PMC2118414 17227914

[41] Lang KS, Recher M, Junt T, Navarini AA, Harris NL, Freigang S.et al. Toll-like receptor engagement converts T-cell autoreactivity into overt autoimmune disease. Nat Med. 2005;11:138–45. http://www.ncbi.nlm.nih.gov/pubmed/15654326. 10.1038/nm1176 15654326

[42] King C, Ilic A, Koelsch K, Sarvetnick N, Jolla L (2004). Homeostatic expansion of T cells during immune insufficiency generates autoimmunity. Cell.

[43] Kousin-Ezewu O, Coles A. Alemtuzumab in multiple sclerosis: latest evidence and clinical prospects. Ther Adv Chronic Dis. 2013;4:97–103. http://www.ncbi.nlm.nih.gov/pubmed/23634277. 10.1177/2040622313479137 PMC3629751 23634277

[44] Costelloe L, Jones J, Coles A. Secondary autoimmune diseases following alemtuzumab therapy for multiple sclerosis. Expert Rev Neurother. 2012;12:335–41. http://www.ncbi.nlm.nih.gov/pubmed/2236433. 10.1586/ern.12.5 22364332

[45] Loh Y, Oyama Y, Statkute L, Quigley K, Yaung K, Gonda E (2007). Development of a secondary autoimmune disorder after hematopoietic stem cell transplantation for autoimmune diseases: role of conditioning regimen used. Blood.

[46] Daikeler T, Labopin M, Di Gioia M, Abinun M, Alexander T, Miniati I.et al. Secondary autoimmune diseases occurring after HSCT for an autoimmune disease: a retrospective study of the EBMT Autoimmune Disease Working Party. Blood. 2011;118:1693–8. http://www.ncbi.nlm.nih.gov/pubmed/21596847. 10.1182/blood-2011-02-336156 21596847

[47] Bastion Y, Coiffier B, Dumontet C, Espinouse D, Bryon PA. Severe autoimmune hemolytic anemia in two patients treated with fludarabine for chronic lymphocytic leukemia. Ann Oncol J Eur Soc Med Oncol. 1992;3:171–2. http://www.ncbi.nlm.nih.gov/pubmed/160609. 1606091

[48] Tosti S, Caruso R, D’Adamo F, Picardi A, Ali Ege M, Girelli G.et al. Severe autoimmune hemolytic anemia in a patient with chronic lymphocytic leukemia responsive to fludarabine-based treatment. Ann Hematol. 1992;65:238–9. http://www.ncbi.nlm.nih.gov/pubmed/1457584. 10.1007/BF01703953 1457584

[49] Myint H, Copplestone JA, Orchard J, Craig V, Curtis D, Prentice AG.et al. Fludarabine-related autoimmune haemolytic anaemia in patients with chronic lymphocytic leukaemia. Br J Haematol. 1995;91:341–4. http://www.ncbi.nlm.nih.gov/pubmed/8547072. 10.1111/j.1365-2141.1995.tb05300.x 8547072

[50] Weiss RB, Freiman J, Kweder SL, Diehl LF, Byrd JC (1998). Hemolytic anemia after fludarabine therapy for chronic lymphocytic leukemia. J Clin Oncol.

[51] Jiang Y, Peng H, Cui X, Zhou Y, Yuan D, Sui X, et al. Autoimmune thrombocytopenia: a complication of fludarabine therapy in the treatment of Waldenstrom’s macroglobulinemia. Int J Clin Exp Med. 2014;7:5937–42. http://www.ncbi.nlm.nih.gov/pubmed/25664138. PMC4307585 25664138

[52] Grimaldi F, Potter V, Perez-Abellan P, Veluchamy JP, Atif M, Grain R, et al. Mixed T cell chimerism after allogeneic hematopoietic stem cell transplantation for severe aplastic anemia using an alemtuzumab-containing regimen is shaped by persistence of recipient CD8 T cells. Biol Blood Marrow Transplant. 2017;23:293–9. http://www.ncbi.nlm.nih.gov/pubmed/27816648. 10.1016/j.bbmt.2016.11.003 PMC5270460 27816648

[53] Bacigalupo A, Socié G, Schrezenmeier H, Tichelli A, Locasciulli A, Fuehrer M.et al. Bone marrow versus peripheral blood as the stem cell source for sibling transplants in acquired aplastic anemia: survival advantage for bone marrow in all age groups. Haematologica. 2012;97:1142–8. http://www.ncbi.nlm.nih.gov/pubmed/22315497. 10.3324/haematol.2011.054841 PMC3409810 22315497

[54] Schrezenmeier H, Passweg JR, Marsh JCW, Bacigalupo A, Bredeson CN, Bullorsky E, et al. Worse outcome and more chronic GVHD with peripheral blood progenitor cells than bone marrow in HLA-matched sibling donor transplants for young patients with severe acquired aplastic anemia. Blood. 2007;110:1397–400. http://www.bloodjournal.org/content/bloodjournal/110/4/1397.full.pdf. 10.1182/blood-2007-03-081596 PMC1939910 17475907

[55] Bacigalupo A, Socié G, Hamladji RM, Aljurf M, Maschan A, Kyrcz-Krzemien S, et al. Current outcome of HLA identical sibling versus unrelated donor transplants in severe aplastic anemia: an EBMT analysis. Haematologica. 2015;100:696–702. http://www.ncbi.nlm.nih.gov/pubmed/25616576. 10.3324/haematol.2014.115345 PMC4420220 25616576

[56] Eapen M, Le Rademacher J, Antin JH, Champlin RE, Carreras J, Fay J, et al. Effect of stem cell source on outcomes after unrelated donor transplantation in severe aplastic anemia. Blood. Am Soc Hematol; 2011;118:2618–21. http://www.ncbi.nlm.nih.gov/pubmed/21677312. 10.1182/blood-2011-05-354001 PMC3167362 21677312

[57] Singhal S, Powles R, Kulkarni S, Treleaven J, Sirohi B, Millar B, et al. Comparison of marrow and blood cell yields from the same donors in a double-blind, randomized study of allogeneic marrow vs blood stem cell transplantation. Bone Marrow Transpl. 2000;25:501–5. http://www.nature.com/articles/1702173. 10.1038/sj.bmt.1702173 10713626

[58] Storek J, Dawson Ma, Storer B, Stevens-Ayers T, Maloney DG, Marr KA (2001). Immune reconstitution after allogeneic marrow transplantation compared with blood stem cell transplantation. Blood.

[59] Bahceci E, Epperson D, Douek DC, Melenhorst JJ, Childs RC, Barrett AJ. Early reconstitution of the T-cell repertoire after non-myeloablative peripheral blood stem cell transplantation is from post-thymic T-cell expansion and is unaffected by graft-versus-host disease or mixed chimaerism. Br J Haematol. 2003;122:934–43. 10.1046/j.1365-2141.2003.04522.x. 10.1046/j.1365-2141.2003.04522.x 12956764

[60] Larosa F, Marmier C, Robinet E, Ferrand C, Saas P, Deconinck E.et al. Peripheral T-cell expansion and low infection rate after reduced-intensity conditioning and allogeneic blood stem cell transplantation. Bone Marrow Transpl. 2005;35:859–68. http://www.ncbi.nlm.nih.gov/pubmed/157651. 10.1038/sj.bmt.1704889 15765116

[61] Jiménez M, Ercilla G, Martínez C. Immune reconstitution after allogeneic stem cell transplantation with reduced-intensity conditioning regimens. Leukemia. 2007;21:1628–37. http://www.nature.com/articles/2404681.pd. 10.1038/sj.leu.2404681 17525730

[62] Le Campion A, Gagnerault M-C, Auffray CD, Bé C, Poitrasson-Riviè Re M, Lallemand E (2009). Lymphopenia-induced spontaneous T-cell proliferation as a cofactor for autoimmune disease development. Blood.

